# Efficacy of drug-eluting bead transarterial chemoembolization in patients with intermediate to advanced hepatocellular carcinoma and its effects on serum γ-glutamyl transferase, calcium-phosphorus metabolism, and hepatic reserve function

**DOI:** 10.12669/pjms.42.4.13779

**Published:** 2026-04

**Authors:** Chen Liu, Yuanlong Zhou, Zan Li, Yuan Wang, Hao Lu

**Affiliations:** 1 Chen Liu, Dept. of Hepatobiliary Surgery, Affiliated Hospital of Hebei University, Baoding 071000, Hebei, China; 2 Yuanlong Zhou, Dept. of Hepatobiliary Surgery, Affiliated Hospital of Hebei University, Baoding 071000, Hebei, China; 3 Zan Li, Dept. of Hepatobiliary Surgery, Affiliated Hospital of Hebei University, Baoding 071000, Hebei, China; 4 Yuan Wang, Dept. of Hepatobiliary Surgery, Affiliated Hospital of Hebei University, Baoding 071000, Hebei, China; 5 Hao Lu, Dept. of Hepatobiliary Surgery, Affiliated Hospital of Hebei University, Baoding 071000, Hebei, China

**Keywords:** Calcium-phosphorus metabolism, Drug-eluting bead, Efficacy, Hepatocellular carcinoma, Transarterial chemoembolization

## Abstract

**Objective:**

To investigate the clinical efficacy of drug-eluting bead transarterial chemoembolization(DEB-TACE) in patients with intermediate to advanced hepatocellular carcinoma(HCC) and its effects on serum γ-glutamyltransferase (GGT), calcium-phosphorus metabolism, and hepatic reserve.

**Methodology:**

A retrospective analysis was conducted on the clinical data of 120 patients with intermediate to advanced HCC treated at Affiliated Hospital of Hebei University between January 2020 to December 2024. Based on the interventional approach, patients were divided into a control group(*n=* 60) and an observation group(*n=* 60). The control group received conventional transarterial chemoembolization (cTACE), while the observation group underwent DEB-TACE. The two groups were compared in terms of treatment response, serum biomarkers, calcium-phosphorus metabolism, hepatic reserve.

**Results:**

The disease control rate was significantly higher in the observation group than in the control group(*P<* 0.05). Serum levels of GGT, α-fetoprotein, and carcinoembryonic antigen decreased in both groups, with significantly greater reductions were observed in the observation group(*P<* 0.05, respectively). Likewise, serum calcium, phosphorus, intact parathyroid hormone, and the calcium-phosphorus product declined in both groups, with significantly lower levels in the observation group(*P<* 0.05, respectively). After treatment, liver function parameters improved in both groups, with more pronounced improvements in the observation group(*P <* 0.05, respectively). The overall incidence of adverse reactions did not differ significantly between the two groups (*P >* 0.05).

**Conclusion:**

DEB-TACE demonstrates multidimensional clinical benefits in the treatment of intermediate and advanced HCC. It offers superior disease control, effectively reduces serum tumor markers, ameliorates calcium-phosphorus metabolic disturbances.

## INTRODUCTION

Hepatocellular carcinoma (HCC) is one of the most prevalent malignancies. The majority of patients are diagnosed at an intermediate or advanced stage, at which point curative surgical resection is no longer feasible.^1^ Although conventional transarterial chemoembolization (cTACE) remains the standard first-line therapy for patients with intermediate to advanced HCC, it has notable limitations, including extensive drug dispersion, incomplete embolization, and a high recurrence rate.^2^ In recent years, drug-eluting bead transarterial chemoembolization (DEB-TACE) has emerged as an innovative locoregional treatment modality for HCC, offering both embolic and sustained drug-release effects.^3^ In DEB-TACE, chemotherapeutic agents are loaded onto biodegradable polymer microspheres and precisely delivered via catheter into the tumor’s feeding artery.

This dual-action mechanism allows for physical occlusion of the tumor’s blood supply, producing an ischemic “starvation” effect, while simultaneously ensuring a controlled, continuous release of the chemotherapeutic agent through ionic interactions on the bead surface.^4^ Serum γ-glutamyltransferase (GGT) serves as a sensitive biomarker of hepatocellular injury and cholestasis, while calcium-phosphorus homeostasis is essential for maintaining skeletal integrity and neuromuscular function. Moreover, hepatic reserve plays a decisive role in determining a patient’s tolerance to interventional therapy and their postoperative recovery capacity. Collectively, these parameters form a core indicator system for evaluating both the efficacy and safety of interventional treatments in HCC.^5^,^6^ Based on this rationale, the present study investigated the comprehensive effects of DEB-TACE on metabolic regulation and hepatic function in patients with intermediate to advanced HCC, aiming to provide a theoretical foundation for optimizing clinical management strategies.

## METHODOLOGY

A retrospective analysis was conducted on the clinical data of 120 patients with intermediate to advanced HCC treated at Affiliated Hospital of Hebei University between January 2020 to December 2024. Patient data including demographic data, diagnosis of breast carcinoma, were retrieved from electronic medical record systems. There were no statistically significant differences in baseline clinical characteristics between the two groups (all P > 0.05) Table-I.

**Table-I:** Comparison of baseline characteristics (*x̅*+*s*, n[%]).

Group	n	Sex		Age (years)	BMI (kg/m²)	Tumor diameter (cm)	Child-Pugh Classification		Comorbidities		
		Male	Female				Class A	Class B	Hypertension	Diabetes	Hyperlipemia
Observation	60	35(58.33)	25(41.67)	57.63±5.48	23.48±2.63	6.67±2.30	21(35.00)	39(65.00)	17(28.33)	13(21.67)	14(23.33)
Control	60	33(55.00)	27(45.00)	57.47±5.40	22.94±2.61	6.38±2.37	19(31.67)	41(68.33)	19(31.67)	12(20.00)	13(21.67)
*χ*²/*t*		0.136		0.168	1.113	0.666	0.150		0.159	0.051	0.048
*P-value*		0.713		0.867	0.268	0.507	0.699		0.690	0.822	0.827

### Ethical approval:

The study was approved by the Institutional Ethics Committee of Affiliated Hospital of Hebei University(No.: HDFY-LL-2022-050; Date: February 21, 2022) and written informed consent was obtained from all participants.

### Inclusion criteria:

- Aged 50~65 years old.
- Meeting the clinical diagnostic criteria for primary liver cancer.^7^
- Diagnosis of intermediate or advanced HCC confirmed by imaging and histopathology.
- Presence of a single tumor lesion.
- Liver function classified as Child-Pugh grade A or B.
- Undergoing treatment for the first time.
- Complete clinical data available.

### Exclusion criteria:

- Severely impaired hepatic function.
- Presence of main portal vein or inferior vena cava tumor thrombus, or extensive extrahepatic metastasis.
- Severe coagulopathy uncorrectable by medical intervention.
- Estimated survival time of less than three months.
- Contraindications to interventional surgery.
- Coexistence of other malignancies and patients with metastatic liver cancer or psychiatric disorders.
- Pregnancy or lactation.

Patients in the control group underwent cTACE. The procedure was performed with the patient in the supine position under local infiltration anesthesia following standard disinfection and draping. Using the Seldinger technique, the right femoral artery was punctured, and a 5F arterial sheath was introduced. A 5F Yashiro or Cobra catheter was then advanced, with the catheter tip positioned at the opening of the celiac trunk. After injecting 10 mL of iohexol (300 mgI/mL) for angiography, the tumor’s feeding arteries were identified, including hepatic arterial branches and potential collateral vessels. Once the target vessel was confirmed, the catheter was superselectively advanced into the main tumor-feeding artery. A chemoembolic emulsion consisting of 100 mL of ultra-fluid iodized oil and 20 mg of pirarubicin was slowly infused through the catheter, followed by an additional 80 mg of cisplatin. Finally, gelatin sponge particles (300-500 μm in diameter) were used to embolize the distal tumor vessels until angiography showed disappearance of tumor staining or a marked reduction in blood flow.

Patients in the observation group underwent DEB-TACE under the same positioning and anesthetic protocol as the control group. Following right femoral artery puncture and placement of a 5F arterial sheath, a 5F RH hepatic catheter was advanced for dual-phase angiography of the celiac trunk and superior mesenteric artery to delineate tumor location, size, and vascular anatomy. Follow-up contrast-enhanced computed tomography (CT) or magnetic resonance imaging (MRI) was performed one month after treatment to evaluate tumor response. If tumor diameter reduction was <30%, retreatment was scheduled 4-6 weeks after the initial procedure, up to a maximum of three sessions.

The dosage of drug-eluting beads was determined according to tumor volume. CalliSpheres® or DC Bead® microspheres (100-300 μm in diameter) were loaded with epirubicin at a ratio of 1-mg of drug per 1-mL of microspheres, with a total loading dose of 50-80 mg adjusted by body surface area. The loaded microspheres were mixed with an equal volume of contrast medium (iohexol) to form a suspension. Using a microcatheter (Progreat® or Renegade®), the suspension was slowly infused into the superselected tumor-feeding artery at a rate of 0.5-1.0 mL/min. The embolization endpoint was defined as complete stasis of the tumor vascular bed with no contrast agent extravasation on angiography. If embolization was incomplete, additional gelatin sponge particles were administered to reinforce the occlusion. Standard repeat treatment was not required; subsequent DEB-TACE was performed only if follow-up enhanced CT showed residual viable lesions, defined as arterial-phase enhancement >15 HU. Both groups were followed for three months after hospital discharge and completed by the same group of surgeons.

### Outcome Measures:

- ***Clinical efficacy:*** At four weeks after treatment, clinical efficacy was evaluated by contrast-enhanced CT or MRI according to the modified *Response Evaluation Criteria in Solid Tumors*.^8^ Complete response (CR) refers to the complete disappearance of target lesions with no evidence of tumor staining or abnormal enhancement on imaging. Partial response (PR) represents a ≥30% reduction in the sum of the longest diameters of target lesions, accompanied by a ≥50% decrease in tumor vascular enhancement. Stable disease (SD) means tumor reduction not meeting PR criteria or tumor enlargement not meeting PD criteria. Progressive disease (PD) denotes a ≥20% increase in the sum of the longest diameters of target lesions, or appearance of new intrahepatic or extrahepatic lesions. The objective response rate (ORR) was calculated as (CR + PR) / n, and the disease control rate (DCR) as (CR + PR + SD) / n.
- ***Serum biomarkers:*** Fasting venous blood samples (2 mL) were collected before treatment and four weeks postoperatively. Samples were centrifuged at 1000 × g for 20 minutes at 4°C to separate serum, which was stored at -20 °C until analysis. Serum GGT, carcinoembryonic antigen (CEA), and α-fetoprotein (AFP) levels were determined via an enzyme-linked immunosorbent assay.
- ***Calcium-phosphorus metabolism:*** Fasting venous blood (2 mL) was collected before treatment and four weeks after treatment. After centrifugation, serum calcium (Ca), phosphorus (P), and intact parathyroid hormone (iPTH) levels were measured using colorimetric assays, and the calcium-phosphorus product was calculated.
- ***Hepatic reserve:*** Fasting venous blood (2 mL) was obtained preoperatively and four weeks postoperatively. After centrifugation, serum alanine aminotransferase (ALT), aspartate aminotransferase (AST), albumin (ALB), and total bilirubin (TBIL) levels were determined using an automated biochemical analyzer.
- ***Adverse reactions:*** Adverse events occurring during hospitalization and within the 3-month follow-up period were recorded, including hemorrhage, pulmonary infection, biliary fistula, ascites, and nausea/vomiting. The overall incidence of adverse reactions was calculated.

### Statistical analysis:

All data were recorded in Excel 2010 and analyzed using SPSS22.0. Measurement data conforming to a normal distribution were expressed as mean ± standard deviation (*x̅*+*s*). Intergroup comparisons were performed using independent-sample t-tests, while intragroup comparisons were analyzed using paired-sample t-tests. Categorical data were expressed as percentages (%) and analyzed using the chi-square (χ^2^) test. A *P-value* <0.05 was considered statistically significant.

## RESULTS

No significant difference in ORR was observed between the two groups (*P >* 0.05). However, the DCR was significantly higher in the observation group than in the control group (*P <* 0.05) Table-II. Before treatment, serum biomarkers did not differ significantly between the two groups (all *P >* 0.05). Four weeks after treatment, serum levels of GGT, AFP, and CEA decreased in both groups, with significantly lower levels in the observation group compared with the control group (*P <* 0.05) Table-III.

**Table-II:** Comparison of clinical efficacy, n(%).

Group	n	CR	PR	SD	PD	DCR	ORR
Observation	60	15(25.00.00)	27(45.00)	15(25.00)	3(5.00)	57(95.00)	42(70.00)
Control	60	9(15.00)	25(41.67)	12(20.00)	14(23.33)	46(76.67)	34(56.67)
*χ^2^*						8.292	2.297
*P-value*						0.004	0.130

**Table-III:** Comparison of serum biomarkers (*x̅*+*s*)

Group	n	GGT (U/L)		AFP (ng/mL)		CEA (ng/mL)	
		Preoperative	Postoperative	Preoperative	Postoperative	Preoperative	Postoperative
Observation	60	215.45±15.22	45.66±5.29^a^	347.53±51.21	157.33±35.29^a^	15.45±1.11	8.66±0.72^a^
Control	60	214.29±15.17	72.18±5.17^a^	349.28±52.33	186.29±37.33^a^	15.37±1.09	9.53±0.81^a^
*t-value*		0.418	27.773	0.185	4.369	0.398	6.218
*P-value*		0.677	<0.001	0.853	<0.001	0.691	<0.001

***Note:***

a P < 0.05 vs. preoperative levels.

Before treatment, no significant differences were found in calcium-phosphorus metabolism between the two groups (*P >* 0.05). After treatment, serum Ca, P, iPTH, and calcium-phosphorus product levels all declined in both groups, with significantly greater reductions observed in the observation group (*P <* 0.05, respectively) Table-IV.

**Table-IV:** Comparison of calcium-phosphorus metabolism (*x̅*+*s*).

Group	n	Ca (mmol/L)		P (mmol/L)		iPTH (pg/mL)		Ca×P product (mg²/dL)	
		Preoperative	Postoperative	Preoperative	Postoperative	Preoperative	Postoperative	Preoperative	Postoperative
Observation	60	2.47±0.48	2.17±0.37^a^	2.11±0.45	1.49±0.31^a^	281.62±35.15	168.31±21.51^a^	66.36±23.55	40.95±13.48^a^
Control	60	2.46±0.45	2.33±0.39^a^	2.13±0.47	1.77±0.33^a^	280.51±34.59	204.28±22.48^a^	66.79±23.66	52.28±16.58^a^
*t-value*		0.118	2.305	0.238	4.789	0.174	8.955	0.099	4.107
*P-value*		0.906	0.023	0.812	<0.001	0.862	<0.001	0.921	<0.001

Note:

a P < 0.05 vs. preoperative levels.

Before treatment, no significant differences were found in hepatic reserve parameters between the two groups (all *P >* 0.05). After treatment, ALT, AST, ALB, and TBIL levels all improved in both groups, with more pronounced improvements in the observation group (*P <* 0.05, respectively) Table-V. The overall incidence of adverse reactions did not differ significantly between the two groups (*P >* 0.05) Table-VI.

**Table-V:** Comparison of hepatic reserve (*x̅*+*s*).

Group	n	ALT (U/L)		AST (U/L)		ALB (g/L)		TBIL (μmol/L)	
		Preoperative	Postoperative	Preoperative	Postoperative	Preoperative	Postoperative	Preoperative	Postoperative
Observation	60	56.15±10.45	43.26±5.29^a^	75.46±15.19	36.45±4.29^a^	35.28±6.15	31.15±3.23^a^	25.41±4.21	15.12±3.11^a^
Control	60	56.33±10.57	51.17±5.66^a^	75.29±15.32	51.24±5.48^a^	35.17±6.07	33.28±3.18^a^	25.57±4.32	17.25±3.19^a^
*t-value*		0.094	7.908	0.061	16.463	0.099	3.640	0.205	3.704
*P-value*		0.925	<0.001	0.951	<0.001	0.922	<0.001	0.838	<0.001

***Note:***

a P < 0.05 vs. preoperative levels.

**Table-VI:** Comparison of adverse reactions, *n*(%).

Group	n	Hemorrhage	Pulmonary infection	Biliary fistula	Ascites	Nausea/vomiting	Overall incidence (%)
Observation	60	1(1.67)	1(1.67)	0(0.00)	0(0.00)	2(3.33)	4(6.67)
Control	60	2(3.33)	2(3.33)	1(1.67)	1(1.67)	3(5.00)	9(15.00)
*χ^2^*							2.157
*P-value*							0.142

## DISCUSSION

In the present study, although no significant difference in ORR was observed between the observation and control groups, the DCR was significantly higher in the observation group. The likely explanation is that drug-eluting beads provide a sustained and controlled release of chemotherapeutic agents, maintaining a steady and effective local drug concentration within the tumor microenvironment, which enhances cytotoxic efficacy and inhibits tumor cell proliferation.^9^ Similarly, Hong S et al. reported that DEB-TACE demonstrated greater advantages in long-term disease control compared with cTACE, supporting the findings of this study.^10^ Regarding serum biomarkers, postoperative levels of GGT, AFP, and CEA in the DEB-TACE group were significantly lower than those in the cTACE group. GGT is not only a sensitive indicator of hepatobiliary dysfunction but has also been recognized in recent years as an independent marker associated with HCC invasiveness and poor prognosis. HCC, particularly at intermediate to advanced stages, presents formidable therapeutic challenges due to its high tumor burden, frequent multicentric growth, and propensity for intrahepatic vascular invasion. These pathological characteristics often limit the efficacy of systemic therapies and are accompanied by considerable toxic and adverse effects.^11^,^12^ DEB-TACE represents a targeted, locoregional therapeutic approach that combines selective vascular embolization with sustained chemotherapeutic drug release. By inducing ischemic necrosis through vascular occlusion and precisely delivering anticancer agents into the tumor-feeding arteries, DEB-TACE exerts a synergistic “starvation and cytotoxic” dual mechanism of action.^13^-^15^

The marked reduction in GGT levels observed in the observation group suggests that drug-eluting beads may achieve more thorough tumor inactivation, effectively reducing the overall tumor burden sustained by residual viable cells and thereby decreasing the secretion of tumor-associated biomarkers.^16^ Likewise, Pérez-López A et al reported significant improvement in serum GGT levels following DEB-TACE, corroborating the findings of this study.^17^ In addition, calcium-phosphorus metabolism was significantly improved in the observation group and was superior to that in the control group. Patients with intermediate to advanced HCC frequently experience disturbances in calcium and phosphorus metabolism because of tumor-related secretion of hormone-like substances or bone metastases.^18^ The metabolic improvement observed in this study may fundamentally stem from effective tumor control achieved by DEB-TACE. As the primary lesion was efficiently suppressed, the secretion of aberrant humoral factors decreased, enabling the gradual restoration of the body’s normal metabolic regulatory mechanisms.^19^ Regarding hepatic reserve, postoperative reductions in ALT, AST, and TBIL levels were significantly greater in the observation group than in the control group, while ALB levels were better preserved.

These findings indicate that DEB-TACE achieves potent tumoricidal effects with comparatively less damage to residual normal hepatic tissue. This advantage may be attributed to the targeted and sustained drug release profile of drug-eluting beads, which markedly reduces systemic exposure and direct cytotoxicity to non-target organs through the circulatory system. In addition, the embolization achieved by DEB-TACE is more precise, minimizing inadvertent occlusion of normal hepatic vessels and thereby providing superior protection of liver function. Chen J et al. similarly demonstrated that DEB-TACE was more effective than cTACE in mitigating postoperative elevations of hepatic enzymes and promoting recovery, consistent with the results of the present study.^20^ No significant difference was observed between the two groups in the overall incidence of adverse reactions, suggesting that while DEB-TACE offers enhanced efficacy, its safety profile remains comparable to that of cTACE. The technological optimization of the procedure thus does not introduce additional or unacceptable risks.

### Limitations:

Nevertheless, this study has several limitations, including a relatively short follow-up period, a modest sample size, and potential confounding variables that could influence the outcomes. Future large-scale, long-term randomized controlled trials are warranted to further validate the efficacy and safety of DEB-TACE and to provide more robust clinical evidence to guide the individualized management of patients with intermediate to advanced HCC.

## CONCLUSIONS

DEB-TACE demonstrates multidimensional clinical advantages in the treatment of intermediate and advanced HCC. It can provide superior disease control, effectively reduce serum tumor burden markers, improve calcium-phosphorus metabolic disturbances, and enhance hepatic reserve, all while maintaining a favorable safety profile.

### Authors’ Contributions:

**CL:** Conceived and designed the study.

**YZ** and **ZL:** Collected the data and performed the analysis. Critical Review

**YW** and **HL:** Writing of the manuscript and is responsible for the integrity of the study.

All authors have read and approved the final manuscript.

## References

[1] Ye T, Shao SH, Ji K, Yao SL (2022). Evaluation of short-term effects of drug-loaded microspheres and traditional transcatheter arterial chemoembolization in the treatment of advanced liver cancer. World J Gastrointest Oncol.

[2] Li H, Zhang X, Zhao W, Cai F, Qin J, Tian J (2023). Efficacy of CalliSpheres®microspheres versus conventional transarterial chemoembolization in the treatment of refractory colorectal cancer liver metastasis. BMC Cancer.

[3] Jin B, Gu Y, Xi S, Liu X, Wu X, Wang X (2024). Efficacy of CalliSpheres®drug-loaded microspheres combined with doxorubicin in hepatocellular carcinoma. Scand J Gastroenterol.

[4] Huang JX, Yang R, Long H, Kong J, Shao GQ, Xiong F (2024). Dual-drug loaded chondroitin sulfate embolization beads enhance TACE therapy for HCC by integrating embolization, chemotherapy, and anti-angiogenesis. Mater Today Bio.

[5] Jia G, Van Valkenburgh J, Chen AZ, Chen Q, Li J, Zuo C (2022). Recent advances and applications of microspheres and nanoparticles in transarterial chemoembolization for hepatocellular carcinoma. Wiley Interdiscip Rev Nanomed Nanobiotechnol.

[6] Zhu HD, Li X, Sun JH, Zhu X, Liu ZY, Li HL (2024). Transarterial Chemoembolization with Epirubicin-Loaded Microspheres for Hepatocellular Carcinoma: A Prospective, Single-Arm, Multicenter, Phase 2 Study (STOPPER Trial). Cardiovasc Intervent Radiol.

[7] Singal AG, Kanwal F, Llovet JM (2023). Global trends in hepatocellular carcinoma epidemiology: implications for screening, prevention and therapy. Nat Rev Clin Oncol.

[8] Zhang B, Yue H (2016). Brief introduction of response evaluation criteria in solid tumors. J Int Oncol.

[9] Chen Q, Shu L, Sun Y, Guo P, Wang D, Sha X (2023). In Vitro Drug Loading, Releasing Profiles, and In Vivo Embolic Efficacy and Safety Evaluation of a Novel Drug-Eluting Microsphere (CalliSpheres). Cancer Biother Radiopharm.

[10] Hong S, Choi WS, Purushothaman B, Koh J, Kim HC, Chung JW (2022). Drug delivery in transarterial chemoembolization of hepatocellular carcinoma: Ex vivo evaluation using transparent tissue imaging. Acta Biomater.

[11] Zhou C, Peng C, Liu F, Xiao J, Li G, Chen C (2023). Evaluation of the efficacy and safety of CalliSpheres®microsphere-transarterial chemoembolization in large hepatocellular carcinoma. J Cancer Res Ther.

[12] Long J, Liu L, Yang X, Lu X, Qin L (2023). Impact of combining Lenvatinib with Transarterial chemoembolization for unresectable hepatocellular carcinoma. Pak J Med Sci.

[13] Zhao G, Liu S, Zhang Y, Zhao T, Wang R, Bian J (2022). Irinotecan eluting beads-transarterial chemoembolization using Callispheres®microspheres is an effective and safe approach in treating unresectable colorectal cancer liver metastases. Ir J Med Sci.

[14] Zhang C, Dai YH, Lian SF, Liu L, Zhao T, Wen JY (2022). Efficacy of transcatheter arterial chemoembolization using pirarubicin-loaded microspheres combined with lobaplatin for primary liver cancer. World J Clin Cases.

[15] Wang Q, Zhu L, Sheng Q (2024). Clinical research progress of callisperes®of drug-loaded microsphere arterial chemoembolisation in the treatment of solid tumors. Discov Oncol.

[16] Pillai K, Ke K, Mekkawy A, Akhter J, Morris DL (2023). Enhancement of treatment efficacy of hepatic tumours using Trans-arterial-chemoembolization. Am J Cancer Res.

[17] Pérez-López A, Martín-Sabroso C, Gómez-Lázaro L, Torres-Suárez AI, Aparicio-Blanco J (2022). Embolization therapy with microspheres for the treatment of liver cancer: State -of-the-art of clinical translation. Acta Biomater.

[18] Zhao GS, Song YX, Sun JB, Liu S, Xu F, Ma J (2023). Efficacy and safety of CalliSpheres®microspheres drug-eluting beads transarterial chemoembolization in GCLM combined trans-arterial infusion therapy for treating primary focus of gastric cancer: a multi-center retrospective study. Expert Rev Anticancer Ther.

[19] Zhang H, Wu C, Chen M, Sun Y, Han J (2023). Drug-eluting bead transarterial chemoembolization (DEB-TACE) versus conventional transarterial chemoembolization (cTACE) in colorectal liver metastasis: Efficacy, safety, and prognostic factors. J Cancer Res Ther.

[20] Chen J, Lai L, Zhou C, Luo J, Wang H, Li M (2023). Safety, efficacy, and survival of drug-eluting beads-transarterial chemoembolization vs. conventional-transarterial chemoembolization in advanced HCC patients with main portal vein tumor thrombus. Cancer Imaging.

